# Use of complementary and alternative medicine (CAM) in Sweden: a cross-sectional survey

**DOI:** 10.1080/02813432.2025.2466180

**Published:** 2025-02-23

**Authors:** Jenny-Ann Brodin Danell, Kathrin Wode, Miek Jong, Agnete Egilsdatter Kristoffersen, Esther van der Werf, Johanna Hök Nordberg

**Affiliations:** ^a^ Department of Sociology, Umeå University, Umeå, Sweden; ^b^ Department of Neurobiology, Care Sciences and Society, Karolinska Institutet, Stockholm, Sweden; ^c^ The National Research Center in Complementary and Alternative Medicine, UiT The Arctic University of Norway, Tromsø, Norway; ^d^ Homeopathy Research Institute, London, UK

**Keywords:** Complementary and alternative medicine, COVID-19, dietary supplements, self care, self-help techniques

## Abstract

**Aims:**

The aim of this study was to describe the prevalence, associations, and reasons for the use of complementary and alternative medicine (CAM) in Sweden, as well as to further explore possible self-perceived outcomes including adverse effects as well as the sources of information used.

**Methods:**

Data were collected by a cross-sectional survey, administered by computer assisted telephone interviews, in June 2020 (*n* = 500), during the first three months of the COVID-19 pandemic. The survey was a modified version of the International Questionnaire to measure use of Complementary and Alternative Medicine (I-CAM-Q) instrument.

**Results:**

63.6% of respondents reported use of CAM. The most common reasons for use were to improve general well-being and/or to treat long-term illness or its symptoms. Very few used CAM to prevent or treat COVID-19. The most used CAMs were natural remedies and dietary supplements (50%), followed by self-help practices (33.2%) and consultation of CAM providers (13%). Women, those of older age (40+), and living in larger cities were more likely to use CAMs compared to the entire sample. Household income or level of education did not predict CAM use. Few adverse effects were reported. The main sources for information were media and the Internet followed by family and friends.

**Conclusions:**

This study contributes with updated knowledge about Swedish citizens’ use of CAM. The results are important to inform health care policy about patterns of CAM use among Swedish citizens.

## Background

Research shows that the use of complementary and alternative medicine (CAM) among the general population in Europe, including Scandinavia, is widespread [1,2]. Since CAM use is associated with benefits and risks, knowledge about characteristics of CAM usage is essential from a public health perspective.

There are several related terms, such as ‘complementary,’ ‘alternative,’ and ‘integrative’ medicine, used to describe approaches that are not typically part of conventional health care and/or may have origins outside Western medical practice. These terms are generally dynamic, evolving over time and across geographical locations [3]. For this study, we use the general and well-established CAM-concept (for a critical discussion, see [4,5]). Moreover, in accordance with the international questionnaire I-CAM-Q [6], we describe the use in three sub-categories: CAM providers (e.g. acupuncturists, massage therapists), natural remedies and dietary supplements, and self-help practices (e.g. meditation, yoga, tai chi, and art therapies).

A European survey reported that an average of 26% of Europeans had used CAM during the last 12 months, ranging from 10 to 40% between countries [2]. The approaches most used include massage, dietary supplements and natural remedies, acupuncture, chiropractic treatment and mind-body practices. Although previous studies about CAM use indicate high prevalence in Sweden, these data are either outdated [7], representing specific regions in Sweden [8,9], or is focused on specific patient or professional groups [10,11].

The use of CAM is most often initiated by users themselves and used as a complement to conventional care [2,12] and more common in women compared to men and among those with higher education. The most reported motives include increased well-being, gaining a sense of control, relieving symptoms and side effects from conventional treatment, and boosting the immune system [2]. Some studies indicate that people with health problems may be more prone to use CAM than those in good health [1,2,9]. It is also suggested that the use of specific CAMs is associated with different health profiles. For example, back- or neck pain has been associated with the use of all kinds of CAMs [2], while depression was only associated with mind-body practices [13]. Moreover, health seeking behavior in general may influence use of CAM [14].

## Aim

The aim of this study was to describe the prevalence, associations, and reasons for use of CAM in Sweden, and further to explore possible self-perceived outcomes including adverse events as well as the sources of information used.

## Methods

### Data collection

For this study, a modified version of the International Questionnaire to measure use of Complementary and Alternative Medicine (I-CAM-Q) was used. The original measure [15] was developed and validated by several research groups (e.g. [16,17]), including adaptations for the Swedish context [9]. In general, the items included are similar to those used in previous studies, although a greater number of natural remedies and dietary supplements were pre-suggested (for full list, see Table 1). In the section, reasons for use, two questions related to COVID-19; ‘for prevention of COVID-19’ and ‘to treat COVID-19-related symptoms’ were added. The data collection was part of a comparative study of prevalence of consultations of health care providers and use of self-management techniques for prevention and treatment of CÒVID-19 related symptoms, in Norway, Sweden and The Netherlands, and was previously published by Kristoffersen et al. [18].

**Table 1.** Use of complementary and alternative medicine (CAM) during the previous three months.

	*n*	% (*N*)	Treatment of acute illness/complaints (with a duration <1 month)	Treatment of chronic illness or its symptoms (with a duration >1 month)	Improving general well-being	Prevent COVID-19	Treat COVID-19	Other reason	Very effective	Moderately effective	Not effective	I don’t know	Yes	No	I don’t know	Internet/media	Licensed health care professionals	CAM providers	Friends and family	Other
Natural remedies and dietary supplements																				
D-vitamin	65	13%	0	21	26	9	2	12	23	16	9	17	0	64	1	25	26	1	12	8
Multivitamin	63	12.6%	1	8	45	4	0	11	15	27	7	14	0	61	2	25	11	1	18	18
Omega 3	60	12%	0	11	39	1	0	10	15	18	9	18	0	59	1	23	12	0	23	10
Ginger	52	10.4%	1	3	33	2	1	0	12	17	3	20	0	51	1	19	6	3	25	6
Magnesium	49	9.8%	1	17	23	2	1	9	19	17	2	11	1	46	2	21	9	3	20	2
C-vitamin, low dose	43	8.6%	0	6	29	5	0	4	8	13	6	16	1	42	0	11	7	2	14	12
Other remedies	38	7.6%	2	10	15	4	1	7	17	6	2	13	3	34	1	16	3	2	17	9
B-vitamin	34	6.8%	1	8	24	0	0	5	11	12	4	7	0	32	2	13	14	0	11	3
Turmeric	32	6.4%	1	3	21	1	0	7	6	9	4	13	0	32	0	16	3	1	10	6
Protein drinks	31	6.3%	0	0	20	0	0	12	11	12	6	2	2	29	0	15	2	1	10	8
Other vitamins and minerals	29	5.8%	0	6	14	1	1	7	5	10	2	12	2	27	1	13	9	0	5	4
Garlic	23	4.6%	0	1	14	2	1	6	7	7	1	8	0	23	0	8	3	0	10	4
Blueberries	22	4.4%	1	1	12	0	0	5	7	3	7	0	0	21	1	8	2	0	8	6
Iron	22	4.4%	0	7	6	0	0	9	7	6	2	7	6	15	1	1	14	1	4	3
Green tea	21	4.2%	0	0	17	1	0	5	3	10	2	6	0	21	0	11	0	1	4	6
Probiotica	21	4.2%	0	2	14	0	0	5	10	5	4	2	0	20	1	7	6	0	4	5
Other dietary supplements	21	4.2%	1	4	9	0	0	10	8	5	2	6	0	20	1	8	3	2	7	4
Zink	20	5%	0	5	13	2	1	1	6	5	1	8	0	18	2	10	2	0	6	3
Herbal tea	17	3.4%	1	3	12	0	0	3	2	10	2	3	0	16	1	6	1	1	5	6
Calcium	15	3%	0	6	9	0	0	1	5	5	1	4	0	14	1	2	8	0	2	3
Cranberries	14	2.8%	2	1	8	0	0	4	5	3	1	5	0	14	0	3	2	0	5	6
Oregano	13	2.6%	0	0	8	0	0	6	2	6	4	1	0	13	0	3	2	0	4	5
C-vitamin, high dose	13	2.6%	0	4	6	3	1	0	4	4	0	5	0	12	1	5	2	1	6	0
Aloe vera	10	2%	1	3	5	1	0	3	3	5	1	1	0	10	0	4	1	0	5	2
Echinacea	5	1%	2	0	1	0	0	2	1	2	1	1	0	5	0	1	0	0	2	2
Rosenrot	5	1%	0	0	4	0	0	1	1	2	1	1	0	5	0	1	2	0	2	2
Ginseng	5	1%	1	0	4	0	0	0	1	3	0	1	0	5	0	3	1	0	1	1
Selenium	4	0.8%	0	0	3	0	0	1	2	1	0	1	0	3	1	2	0	0	2	0
Homeopathic remedies	3	0.6%	0	1	2	0	0	1	3	0	0	0	0	3	0	1	0	1	2	0
Anthroposophic remedies	0	0%	0	0	0	0	0	0	0	0	0	0	0	0	0	0	0	0	0	0
Self-help practices																				
Breathing exercises	65	13%	3	10	40	6	1	11	38	17	6	4	2	63	0	32	20	8	14	9
Yoga	47	9.4%	0	8	42	0	0	3	27	17	3	0	0	46	1	21	3	7	16	9
Mindfulness	39	7.8%	0	5	18	0	0	5	24	11	3	1	2	37	0	16	14	3	15	10
Other self-help techniques	38	7.2%	0	6	24	2	0	10	28	7	1	0	5	31	0	8	9	1	10	14
Meditation	36	7.2%	0	4	30	0	0	3	26	9	1	0	2	33	1	22	3	3	9	6
Relaxation	36	7.2%	1	7	24	0	1	7	22	11	1	2	2	32	2	12	9	5	10	10
Prayer for own health	24	4.8%	2	4	15	5	1	4	18	3	2	1	1	21	2	5	0	1	10	15
Visualization	13	2.6%	0	1	8	0	0	6	8	4	0	1	1	12	0	7	0	3	3	5
Qi Gong	7	1.4%	0	1	5	0	0	1	6	1	0	0	0	7	0	3	1	1	3	3
Tai Chi	2	0.4%	0	0	2	0	0	0	2	0	0	0	0	2	0	1	0	1	2	1
Consultation with CAM providers																				
Massage therapist	31	6.2%	4	10	16	0	0	3	16	13	1	1	2	29	0	6	5	2	11	8
Psychotherapist	14	2.8%	1	9	4	0	0	1	9	3	1	1	0	14	0	1	4	0	4	5
Naprapath	10	2%	1	6	0	1	0	3	7	3	0	0	1	9	0	3	2	0	2	3
Chiropractor	8	1.6%	4	3	1	0	0	0	3	3	1	1	0	8	0	3	4	0	2	0
Acupuncturist	7	1.4%	1	4	0	0	0	2	5	1	1	0	2	5	0	2	2	0	3	1
Coach	5	1%	0	2	2	0	0	1	5	0	0	0	0	5	0	2	0	0	3	1
Food/nutrition specialist	3	0.6%	0	1	2	0	0	0	0	3	0	0	1	2	0	0	2	0	1	0
Osteopath	2	0.4%	0	2	0	0	0	0	2	0	0	0	0	2	0	2	0	0	0	0
Spiritual guide	2	0.4%	0	0	1	0	0	1	1	0	1	0	0	2	0	0	0	0	2	0
Zone therapist	1	0.2%	0	1	0	0	0	0	1	0	0	0	0	1	0	0	0	0	1	0
Traditional Chinese medicine	1	0.2%	0	1	0	0	0	0	0	0	0	1	0	1	0	1	0	0	0	0
Homeopath	1	0.2%	0	1	0	0	0	0	1	0	0	0	0	1	0	0	0	0	0	1
Kinesiologist	0	0%	0	0	0	0	0	0	0	0	0	0	0	0	0	0	0	0	0	0
Ayurvedic health counselor	0	0%	0	0	0	0	0	0	0	0	0	0	0	0	0	0	0	0	0	0
Anthroposophic health counselor	0	0%	0	0	0	0	0	0	0	0	0	0	0	0	0	0	0	0	0	0

The data collection was conducted by computer assisted telephone interviews, between 15 June 2020 and 23 June 2020, in collaboration with Ipsos (Stockholm, Sweden). The target sample was set to 500 individuals, representing the adult Swedish population of 10.4 million inhabitants at the time of the study. The sample used was stratified by sex, age and region of residence to increases the representativeness of the target population. To ensure sufficient study power for representing the adult Swedish population, we calculated the minimum required sample of 385 based on a 5% margin of error, a 95% confidence level and an assumed response distribution of 50% (https://www.idsurvey.com/en/sample-size-calculator/). Recognizing that a larger sample size can reduce sampling error and improve the likelihood of accurately reflecting the population characteristics, we increased the sample size to 500. In total, seven attempts were made to reach 6500 individuals. 5571 were not reachable, 429 denied participation and 500 accepted invitation. The response rate for the entire sample was 7.7%. Among the individuals who were reachable, the response rate was 53.8%.

### Measures

Respondents were asked about the following personal characteristics; sex (female, male), living conditions (living on her/his own, married or living with partner, living with parents or sibling, living with others) and residence (living in the countryside, small city, large city). Age was obtained by an open question, and the responses were merged in four categories (15–24 years, 25–39 years, 40–59 years, 60+ years). Level of education was measured by four categories (elementary school, secondary school, university education up to four years, university education more than four years). Household income was measured by 14 categories, later merged into low household income (<100,000–399,000 SEK), middle household income (400,000–799,000 SEK), high household income (+800,000 SEK), don’t want to report and don’t know.

The questionnaire was divided into three sections: natural remedies and dietary supplements, self-help practices, and consultation with CAM providers. The respondents were asked if they had used one or more of the options (see lists in Table 1), during the previous three months. If yes, they were asked about motives (treatment of acute illness/complaints with a duration <1 month, treatment of chronic illness or its symptoms with a duration >1 month, improving general well-being, to prevent COVID-19, to treat COVID-19, other reasons), effects (very effective, moderately effective, not effective, don’t know), side effects (yes, no, don’t know) and from where/who they got information (the Internet/media, authorized health professional, CAM provider, family/friends, other). The options other/other reasons were open-ended questions.

Over-all use of CAM was measured by combining the variables consultation with CAM providers, natural remedies and dietary supplements, and self-help practices. The results were merged into four categories; non-users, use of 1–2 CAMs, use of 3–5 CAMs, and use of 6+ CAMs.

### Analysis

Descriptive statistics were carried out using Statistical Package for Social Sciences (SPSS), v 27 (SPSS Inc., Chicago, IL). Pearson’s Chi-Square test was performed to identify significant differences regarding sociodemographic variables (age, education and household income) between men and women and between users and non-CAM users. Significance level was set to *p* < .05. It should be noted that this type of test is designed for hypothesis-driven research and is associated with several well-known issues, such as overestimation of clinically relevant results [19]. Therefore, significant differences should be interpreted with caution. To support interpretation, we present the results along with actual *p* values [20] and detailed data.

When not stated otherwise, the percentages in the presentation below refer to the whole sample of respondents. Since the respondents could report use of several CAMs, the number of cases in different categories is presented when relevant.

## Results

### Basic characteristics of the respondents

An overview of the basic characteristics of the respondents is presented in Table 2. The sample consisted of slightly more women (50.8%) than men (49.2%). More men were represented in the youngest age group while fewer men were in the oldest. The opposite pattern was observed for women with fewer women in the youngest age group and more in the oldest. Most respondents had a secondary (41.6%) or university education (27.2% up to four years, 18.4% more than four years). In general, women were more highly educated than men. 43.8% of the respondents reported middle or high household incomes, but almost 29% did not know or didn’t want to report their incomes. A total of 55.6% of the respondents were married or living with a partner, 30.8% were living on their own. More of the women were married or living with a partner, and more of the men were living on their own, with parents/siblings, or with others. Of all respondents, 34.4% lived in a large city, 35.6% in a small city, and 30% in the countryside. For the whole sample, differences between men and women were significant regarding age (*p* = .002), education (*p* = .018) and living conditions (*p* = .046).

**Table 2.** Basic characteristics of the respondents.

	Total		Men		Women		*p* Value
	*N*	%	*n*	%	*n*	%	
Gender	500	100	246	49.2%	254	50.8%	
							.002
Age group							
15–24 years	77	15.4%	52	21.1%	25	9.8%	
25–39 years	105	21.0%	51	20.7%	54	21.3%	
40–59 years	152	30.4%	75	30.5%	77	30.3%	
60+ years	166	33.2%	68	27.6%	98	38.6%	
Education							.018
Primary school	64	12.8%	38	15.4%	26	10.2%	
Secondary education	208	41.6%	113	45.9%	95	37.4%	
College/university (up to 4 years)	136	27.2%	57	23.2%	79	31.1%	
College/university (more than 4 years)	92	18.4%	38	15.4%	54	21.3%	
Household income							.08
Low (<100,000–399,000 SEK)	137	27.4%	76	30.9%	61	24.0%	
Middle (400,000–799,000 SEK)	145	29.0%	78	31.7%	67	26.40%	
High (+800,000 SEK)	74	14,8%	30	12.2%	44	8.80%	
Don’t report	24	4,8%	12	4.9%	12	2.40%	
Don’t know	120	24.0%	50	20.3%	70	27.60%	
Area of living							.097
Large city	172	34.4%	77	31.3%	95	37.4%	
Small city	178	35.6%	99	40.2%	79	31.1%	
Rural area	150	30.0%	70	28.5%	80	31.5%	
Living conditions							.046
Living on my own	154	30.8%	81	32.9%	73	28.7%	
Married/living with partner	278	55.6%	123	50.0%	155	61.0%	
Living with parents/siblings	39	7.8%	23	9.3%	16	6.3%	
Living with others	29	5.8%	19	7.7%	10	3.9%	

### Use of conventional health care

The respondents were asked if they had visited conventional healthcare providers during the previous three months. A total of 32.6% had visited medical doctors within the last three months, most to treat acute illness/complaints or chronic illness or its symptoms. 7.8% had consulted ‘other providers,’ such as nurses, physiotherapists, dentists, and psychologists. Since most of these providers are part of conventional health care in Sweden, it is reasonable to classify them as such and not as part of CAM. The majority (65.3%) found conventional health care ‘very effective,’ and most users (86.6%) reported no negative side effects. Significantly (*p* = .02) more women than men had visited conventional healthcare providers.

### Use of CAM

A total of 63.6% of the respondents had used CAM, in terms of natural remedies/dietary supplements, self-help practices or consulting CAM providers during the previous three months. More women (70.5%) than men (56.5%) were found in this group, and more CAM users were found in the two oldest age groups (40–59 years, 60+ years). Slightly more non-users lived in the countryside, and those who had used 1–2 CAMs were relatively equally distributed. Extensive users more often lived in larger cities. Statistically significant differences were found for gender (*p* = .001), age group (*p* = .04) and residency (*p* = .014) for the overall use of CAM. There were no significant differences for household income and level of education.

As presented in more detail below, the most frequently used CAMs were natural remedies and dietary supplements (50%), followed by self-help practices (33.2%), and consultation of CAM providers (13%). It was most common to have used one or two types of CAMs (33%), 18.4% had used 3–5 CAMs, and 12.2% used six or more.

### Natural remedies and dietary supplements

Half of the respondents (50%) had used natural remedies or dietary supplements during the previous three months, and there were significantly (*p* = .001) more women (55.9%) than men (43.9%) among the users. Regarding other background variables, only residency showed significant differences (*p* = .007) with more non-users living in the country-side, and moderate users (1–2 CAMs) living in large cities.

The most used remedies and dietary supplements (see Table 1) were D-vitamin, multivitamin, Omega 3 and ginger, and the most common motive for using these was to improve general well-being. A majority of the users found natural remedies or dietary supplements ‘very effective’ or ‘moderately effective’ but compared to use of other CAM-types more respondents in this group reported that the treatments were ‘not effective’ or that they ‘don’t know’ about the effects. Few respondents reported negative side effects (15 cases). Compared to the other types of CAM use, most reports related to prevention and treatment of COVID-19 (a total 42 cases) were found in this group. Eleven participants reported using vitamin D for this purpose. Regarding information, the most common source was the Internet and media (281 cases), followed by family and friends (244 cases). Natural remedies and dietary supplements were recommended by authorized health care providers to a relatively large extent (151 cases).

### Self-help practices

A total of 33.2% of the respondents reported using self-help practices during the last three months. There were significantly (*p* = .002) more women (40.6%) than men (25.6%) among the users. Furthermore, there were significant differences regarding age group (*p* = .012) and education (*p* = .020). For example, it was most common for non-users to have primary school or secondary school as highest education. Among moderate users, it was most common to have university education up to four years or more than four years. Regarding age, most non-users belonged to the youngest or oldest age groups. There were no significant differences regarding living conditions and income.

The most reported self-help practices were breathing exercises, yoga and mindfulness. Many of the users had used one or two self-help practices, and most reported that they found the practices ‘very effective’ or ‘moderately effective.’ Few reported negative side effects (15 cases). The most common sources of information were the Internet and media (127 cases), followed by family and friends (92 cases).

### Consultation of CAM providers

As presented above, Table 1, 13% of the respondents consulted a CAM-providers during the previous three months. Among the users, it was most common to have consulted one or two providers. The only background variable with significant differences was age. More non-users were found in the younger and older age groups, and more users were found in the middle age group (40–59 years).

The most used modalities were massage therapy and psychotherapy, both of which are generally accepted in Swedish society and to some extent integrated into official health care. The most common motive was to treat chronic illness or its symptoms. A majority of those who used consultation with CAM providers reported that they found the treatment ‘very effective’ or ‘moderately effective.’ Very few reported ‘not effective’ or that they ‘don’t know’ about the effects. Furthermore, few respondents reported negative side effects (in total six cases). Regarding sources of information, the most used source was family and friends (29 cases), followed by the Internet and media (20 cases).

## Discussion

In summary, 63.6% of respondents reported using some type of CAM in the past three months. The most common motives for use were improving general well-being and/or treating long-term illness or its symptoms. Very few respondents used CAM to prevent or treat COVID-19. The most used CAMs included natural remedies and dietary supplements (50%), followed by self-help practices (33.2%), and consultations with CAM providers (13%). Most respondents reported using one or two types of CAM during the period. Women, individuals aged 40 years and older, and those living in larger cities were more likely to use CAM compared to the entire sample. Household income and education level did not predict CAM use. Few adverse effects were reported. The main sources of information about the used CAM were media, the Internet, and family and friends.

The relatively high CAM utilization observed in studies using the I-CAM-Q measure (e.g. [6,9,18]), including this study, compared to studies using other measurements (e.g. [1,2,8]), is likely due to the comprehensive list of probing questions featuring specific CAMs, as detailed in Table 1.

In line with most previous research on the topic (e.g. [1,2]), this study found CAM usage to be more common among women than men. Although this may reflect higher rates of unmet health care needs among women compared to men [1,21] found that these factors could not describe this association. In line with research from the US [22], they argue that the popularity of CAM usage is likely also to reflect personal values. Like the results of Gildayal et al. [23], this study shows that most CAM users experienced these methods as effective, and few adverse effects were reported (cf. [24]).

For most of the CAM usage reported in this study, conventional health care was seldom reported to be the primary source for information. Similar to what has been found in other studies [6,11], the primary sources were the Internet, media, family and friends, despite an explicit wish among many CAM users to have a dialogue with conventional health care [11].

The results of this study must be interpreted with caution. Although the study population was calculated to be large enough to resemble the general adult population in Sweden, the response rate for the entire sample was only 7.7%, including a large proportion of non-reachable individuals (5571 out of 6500). Among those who were reachable, the response rate was 53.8%. Despite the sample was stratified by sex, age and region of residence to increase the representativeness of the target population, the low response rate may have resulted in a non-representative sample. Additionally, there is missing information regarding the respondents’ self-reported incomes, making it difficult to assess such demographic imbalances. Detailed results, such as the usage of particular remedies or self-help practices, should be interpreted carefully, as they are, in most cases, based on very few respondents. Similarly, significant differences should not be overestimated.

It is unclear whether the willingness to participate in the study was influenced by its timing during the early phase of the pandemic. Nonetheless, the overall CAM usage among the Swedish population reported in this study resembles previously reported results from similar questionnaires conducted before and during the initial months of the COVID-19 pandemic in the Nordic countries [6,9,18,25,26]. This suggests that the reported CAM usage during this period did not significantly differ from non-pandemic conditions.

## Conclusions

This study makes an important contribution with updated knowledge about Swedish citizens’ use of CAM. The study highlights that CAM use is common among respondents, with natural remedies and dietary supplements being the most frequently used. Usage is driven primarily by the desire to improve general well-being and/or manage long-term illnesses. Women, older individuals and urban residents are more likely to use CAM, while income and education levels do not predict usage. Information about CAM was primarily from media, the Internet and social networks, and few adverse effects are reported. These results are important to inform health care policy about patterns of CAM use among Swedish citizens.
